# Two-port approach for fully thoracoscopic right upper lobe sleeve lobectomy

**DOI:** 10.1186/1749-8090-8-99

**Published:** 2013-04-17

**Authors:** Wenjie Jiao, Yandong Zhao, Tao Huang, Yi Shen

**Affiliations:** 1 Department of Thoracic Surgery, the Affiliated Hospital of Medical College, Qingdao University, 16 Jiangsu Road, Qingdao 266003, China

**Keywords:** Lobectomy, Thoracoscopy, Lung cancer surgery, Surgery/incisions, Minimally invasive surgery

## Abstract

This report describes a case report of a minimally invasive technique for VATS right upper sleeve lobectomy with a two-port approach. To our knowledge it is the first report of this kind. A 50-year-old man with a pulmonary nodule occluding the orifice of the right upper lobe bronchus was referred to our department. Dissection, stapling the right upper lobe pulmonary vessels and anastomosis between the right intermediate and the right main bronchus were performed via the two port. To deal with blocking of pulmonary artery and obtain a satisfactory exposure and manipulating space in the course of bronchial anastomosis were the key points. Intraoperative blood loss was 150 ml and total operative time was 220 minutes. The postoperative course was uneventful. Chest X-rays showed no sign of atelectasis. Postoperative histopathological examination revealed that the tumor was T3N0M0 squamous cell carcinoma. The patient was discharged from hospital on postoperative day 9 without any complications. We conclude that video-assisted thoracoscopic sleeve lobectomy with mediastinal dissection by two-port approach is feasible and convenient.

## Background

Video-assisted thoracoscopic surgery is appealing alternative to thoracotomy due to numerous advantages [1]. However, only a few reports exist on VATS sleeve lobectomy [2-4]. In most of these, bronchial anastomoses were accomplished in an open surgical technique through a minithoracotomy. This report describes a minimally invasive technique for VATS right upper sleeve lobectomy with a two-port approach.

## Case presentation

**Figure 1:**
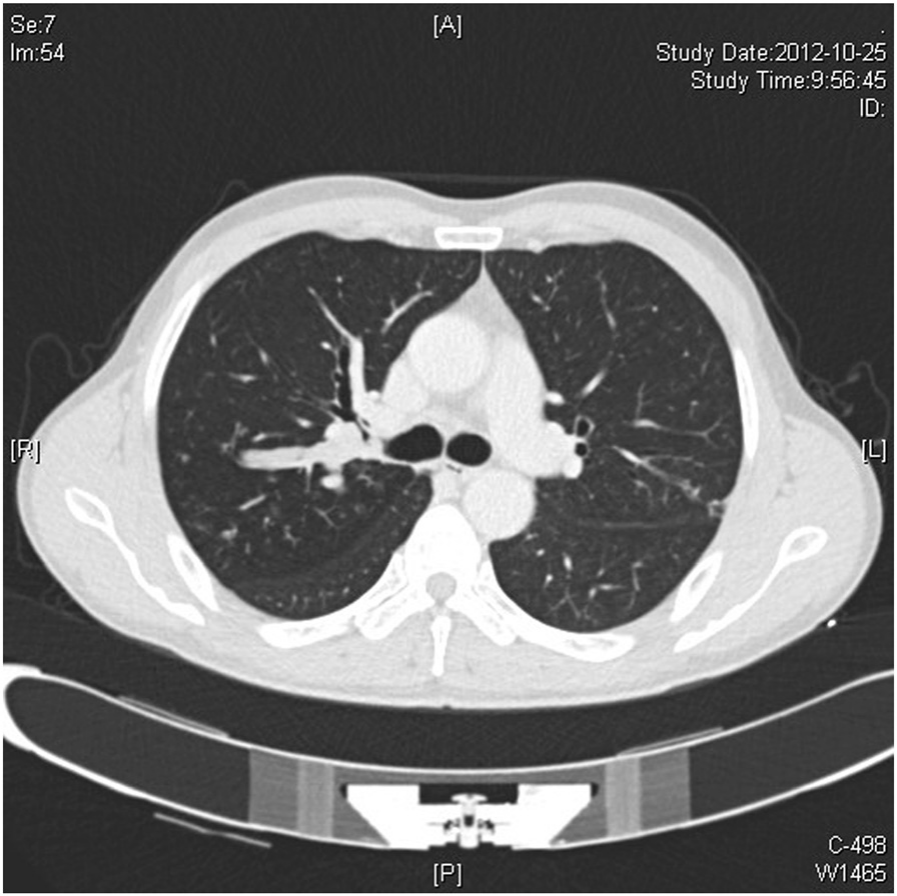
A computed tomographic image had shown the tumor originating from the right upper lobe bronchus (lung windows).

**Figure 2:**
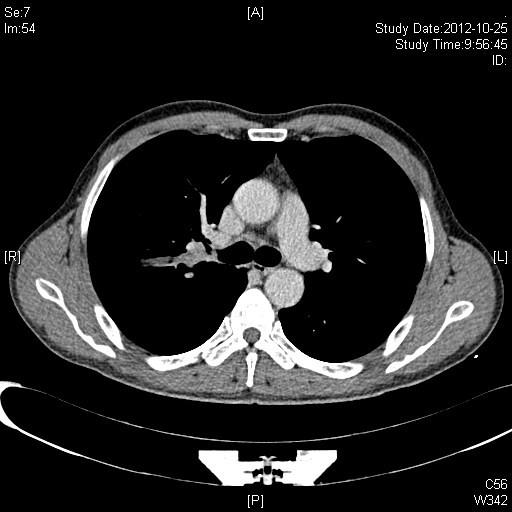
A computed tomographic image had shown the tumor originating from the right upper lobe bronchus.

A 50-year-old male (Chinese, ethnic Han) consulted for cough and hemoptysis. A computed tomography (CT)-scan revealed a mass in the right upper lobe. The bronchoscopy showed a mass occluding the orifice of the right upper lobe bronchus (Figures 1 and 2). The biopsy revealed a squmous cell carcinoma. Respiratory test function were normal. The patient was proposed for video-assisted thoracoscopic surgery (VATS).

**Figure 3:**
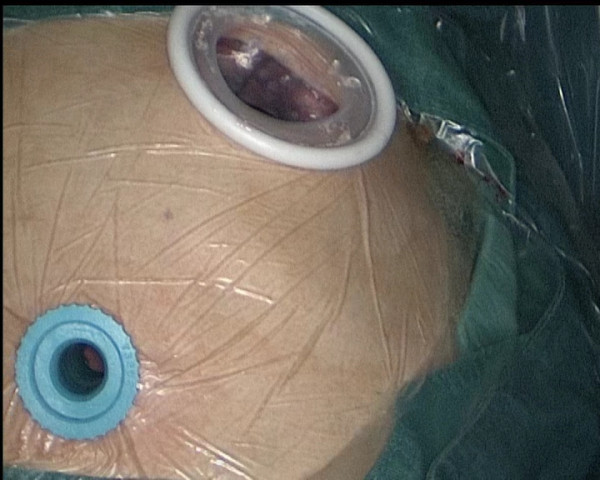
Two port: camera port and utility port.

The patient was placed in a left lateral decubitus position and double-lumen intubation. The first 1.5-cm incision is performed in the 7th intercostal space in the mid-axillary line, and is used mainly for introducing a 10-mm 30^o^ thoracoscope. The second incision, 4 cm long, was made in the 4th intercostal space in the anterior position just between latissimus dorsi and pectoralis major (Figure 3). We completed whole operation without visual access through the incision and without rib spreading. We use electronic hook, curved suction apparatus and other conventional long instruments, combined with thoracoscopic equipment.

**Figure 4:**
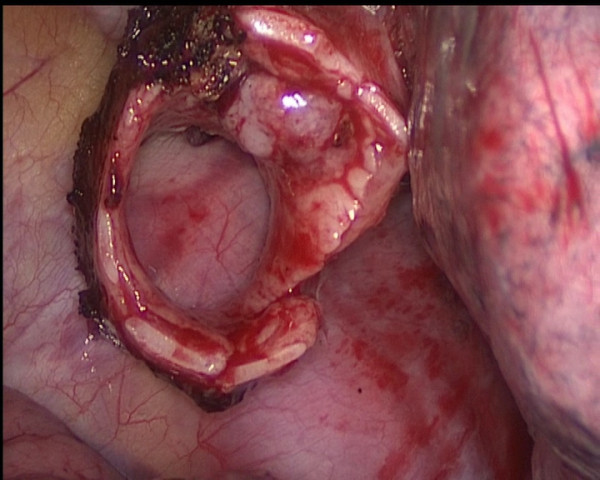
The tumor had been shown originating from the right upper lobe bronchus.

The first action is to retract the lung up and forthwards by orbicular-ovate grasping forceps using the utility port. After adequate exposure of the lung, we begin the inferior pulmonary ligament and posterior mediastinal visceral pleura on dissection through the utility port. Next, we retracted the lung down and backwards, and dissect the right upper lobe pulmonary veins. And then, the 30° camera and the endostaplers are exchanged from one incision to the other for the resection of the right upper lobe pulmonary veins. Next, the anterior trunk and posterior ascending branch of the pulmonary artery were dissected and stapled. The posterior fissure was completed, followed by the stapling of the anterior fissure. Subcarinal lymphadenectomy is then meticulous done in order to identify and handle the right main bronchus and the intermediate bronchus. A long scissors through the utility incision is used to transect the bronchus proximal and distal to the tumor. The length of resected bronchus was about 2-cm (Figure 4). The lobe is completely disconnected, it is placed in a latex glove and brought out through the utility incision. The specimen was sent for frozen section, which confirmed free margins.

**Figure 5:**
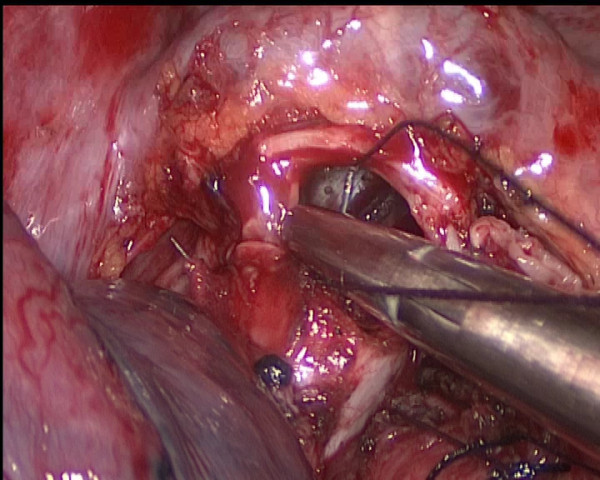
Running suture in the posterior row.

The bronchial reconstruction with the anastomosis between the right main bronchus and the intermediate bronchus was performed, and the two ends of the bronchus are then brought together using running sutures with two 3–0 Vicryl (Ethicon). According to our past experiences and habits, the anastomosis methods and materials were the same as sleeve lobectomy by thoracotomy. We oppressed the right pulmonary artery by long curved sunction apparatus in order to get a good visual field and manipulating space. The posterior row is completed first using one 3–0 Vicryl (Ethicon) from the site of the two corners of the pars cartilaginea to the pars membranacea (Figure 5), followed by the anterior row using other. Finally, the two line were tied a knot at the pars cartilaginea. Initially, a distinct air leakage by the side of the knot was detected when setting a normal saline and 20 cmH_2_O ventilation positive end-expiratory pressure. It was closed with one additional interrupted suture. A complete mediastinal lymph node dissection is then dissected thoracoscopically.

The chest is then irrigated, and the anastomosis is checked for an air leak under water. A single chest tube was placed and the incisions were closed. The total surgery time was 220 min, including 60 minutes for performing the anastomosis. Estimated intraoperative blood loss was 150 mL.

**Figure 6:**
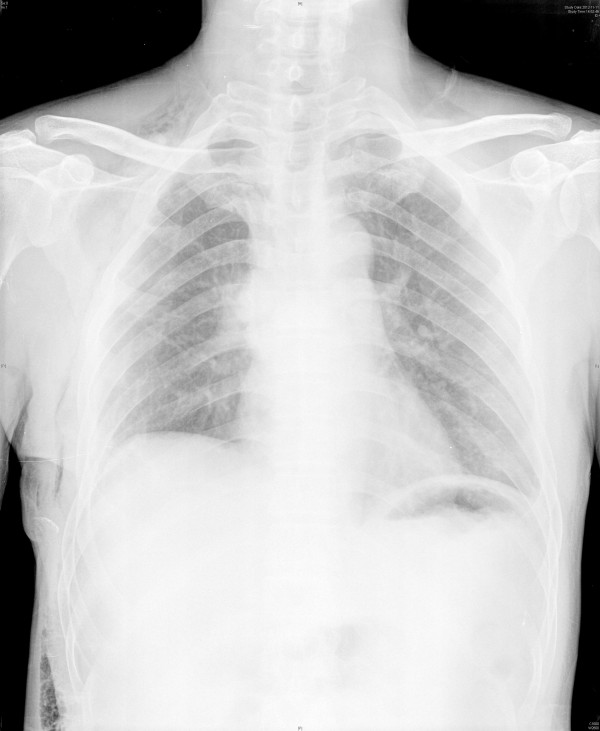
Chest X-rays showed no unnormal sign on postoperative day 7.

The patient was extubated in the operating room and brought to recovery room. Postoperative chest X-rays showed no sign of atelectasis (Figure 6). The chest tube was removed on postoperative day 4. The patient was discharged home on postoperative day 9 with no complications in the chest X-ray and with good functional recovery. The pathological examination revealed a 2-cm squamous cell carcinoma with no lymph node involvement (a total of sixteen lymph nodes in the station of 2,4,7,10,11 were studied).

## Discussion

Most of the thoracic surgeon performed VATS lobectomy using 3 to 4 ports, including a utility incision measuring about 3–5 cm and 2 to 3 other ports. Actually, the lobectomy may be successfully carried out using only two ports as the technique described by Burfeind and D’Amico [5]. There are few teams in the world that carry out this procedure and the largest series of this procedure is at Duke Medical Center D’Amico’s group which reports on an extensive series of 500 cases [6,7]. Two-port lobectomies are a consequence of greater skills acquired with experience. Borro and his colleagues reported that the realisation of two ports should neither prolong estimated operative time nor hinder cleaning of the lymph nodes, nor increase the likelihood of surgical or postoperative complications [8].

Sleeve lobectomy has been almostly absolute indication for conversion to thoracotomy until recently. The anastomosis was created partially or totally under direct vision. The first case report on a VATS sleeve lobectomy was given by Santambrogio and colleagues in 2002 [2]. The maximum report with 13 patients who underwent VATS sleeve lobectomy was published in 2008 by McKenna and colleagues [3]. Schmid and his colleagues report on a combined robotic and VATS approach for a true minimally invasive right upper sleeve lobectomy [4].

We began performing fully VATS lobectomies in June 2009. Up to October 2012, we undertook 260 major pulmonary resections by VATS, including lobectomy, bilobectomy, segmentectomy and sleeve lobectomy. The first 90 cases were operated on using three ports, but since March 2010, we started to perform fully VATS lobectomies and complete lymph node dissections using only two-port in almost all cases. In our literature review, we have found no reports of sleeve lobectomy performed through a two-port.

This case shows the technical feasibility and safety to do a VATS sleeve lobectomy with only two-port. Theoretically, this approach may produce less pain and less immunologic response due to less invasive. Moreover, obviating the posterior incision may reduce the possibility of hemorrhage of chest wall muscles. Further studies will be required to identify any quantifiable advantage of two-port approach.

## Conclusion

Although an advanced technique is required for full thoracoscopic surgery by two-port, it is feasible to perform VATS sleeve lobectomy via this approach.

## Consent

Written informed consent was obtained from the patient for publication of this report and any accompying images. A copy of the written consent is available for review by the Editor-in-Chief of this journal.

## Competing interests

The authors declare that they have no competing interests.

## Authors’ contributions

All the authors participated in the treatment of this patient. WJ performed the operation and drafted the manuscript. YZ, TH and YS helped to draft the manuscript. All authors read and approved the final manuscript.

## References

[B1] Ettinger DS Bepler G Bueno R National Comprehensive Cancer Network (NCCN): non-small cell lung cancer clinical practice guidelines in oncology J Natl Compr Canc Netw 2004 2 94 124 19777701 10.6004/jnccn.2004.0010

[B2] Santambrogio L Cioffi U De Simone M Rosso L Ferrero S Giunta A Video-assisted sleeve lobectomy for mucoepidermoid carcinoma of the left lower lobar bronchus: a case report Chest 2002 121 635 636 10.1378/chest.121.2.635 11834681

[B3] Mahtabifard A Fuller CB McKenna RJ Jr Video-assisted thoracic surgery sleeve lobectomy: a case series Ann Thorac Surg 2008 85 S729 S732 10.1016/j.athoracsur.2007.12.001 18222205

[B4] Schmid T Augustin F Kainz G Pratschke J Bodner J Hybrid video-assisted thoracic surgery-robotic minimally invasive right upper lobe sleeve lobectomy Ann Thorac Surg 2011 91 1961 1965 10.1016/j.athoracsur.2010.08.079 21619991

[B5] Burfeind WR D’Amico TA Thoracoscopic lobectomy Op Tech Thorac Cardiovasc Surg 2004 9 98 114 10.1053/j.optechstcvs.2004.05.002

[B6] Onaitis MW Petersen RP Balderson SS Thoracoscopic lobectomyisa safe andversatile procedure: experience with 500 consecutive patients Ann Surg 2006 244 420 425 16926568 10.1097/01.sla.0000234892.79056.63 PMC1856541

[B7] Villamizar N Darrabie M Burfeind W Thoracoscopic lobectomy is associated with lower morbidity compared with thoracotomy J Thorac Cardiovasc Surg 2009 138 419 425 10.1016/j.jtcvs.2009.04.026 19619789

[B8] Borro JM Gonzalez D Paradela M The two-incision approach for video-assisted thoracoscopic lobectomy: an initial experience Eur J Cardiothorac Surg 2011 39 120 126 Epub 2010 Jun 16 10.1016/j.ejcts.2010.05.010 20558077

